# Annotations

**Published:** 1902-08-02

**Authors:** 


### Annotations.

### Ice Charities.

In New York there is a summer charity which has not yet, so far as we know, been started here. It is the supplying of ice gratuitously to the sick poor, and to families where there are young children. Ice is a necessary of life in American households. This may be to some extent a matter of custom in a country where water is hardly drunk unless it is iced, where fruit is kept chilled on ice until it is about to be eaten, and where the refrigerator is an almost invariable part of the household furnishing. The prospects of the ice crop are discussed in the newspapers in the same way as the prospects of the corn crop, and the big white sheds on the banks of the Hudson and other rivers, where the ice is stored until wanted, though they do not add to the beauty of the scenery, show unmistakably the important position which the daily supply of ice holds in American life. To be without ice there is much like being without tea in this country; it is doing without a comfort which is so common as to be almost a necessity. Our climate does not demand that ice should have such a prominent place in our homes as it does in those of our trans-Atlantic cousins, but there can be no doubt of the comfort and convenience of ice in hot weather. And the place where it would be most useful is just where it is least likely to be found—in the crowded homes that line the narrow airless streets of the poorer districts of our towns. A morsel of ice to make the drink of water really cold and refreshing would be a boon to many a fevered creature, ice enough to keep the milk sweet in the close room might save many a child's life. There is probably no need of special ice charities in this country, but there is a need that ice should be a thing which charity might give to its recipients far oftener than is now done.

### Cures for Inebriates.

A correspondent writes to us a sad account of a man, apparently in good position, who came over from South Africa to undergo an advertised treatment for inebriety, and, being placed in rooms without any attendant while in a condition of extreme despondency, cut his throat. Our correspondent, who is a lady and evidently full of sympathy, says "had the poor lonely stranger had a sympathetic friend he would probably have been well now instead of in a suicide's grave. It is pathetic that his last words were that he was 'so lonely and had not a soul to speak to.'" She goes on to say that the system adopted by advertising inebriety curers, according to which their "patients" are placed in boarding houses or in private rooms and left to wander about without any control whatever, involves enormous risks, and in all of this we quite agree. When, however, she adds that this course "would not be tolerated by the medical authorities if the facts were fully known," we can but say that she has an exaggerated idea of the powers of "the medical authorities," whoever they may be. It is, unfortunately, the fact that there are no medical authorities which have the slightest power to prevent anybody putting himself in the hands of any quack to whom he may choose to entrust his health or his property. We quite agree with our correspondent when she says that many of the "patients" alluded to are of "unsound mind," and obviously the fact that they are not entirely responsible for their actions only adds to the scandal of a system of quackery being allowed to prey upon such people, but, as things are at present, we see no means of preventing it. So long as charlatans keep within the four corners of the law and are backed up by clerics and other fashionable people they can do much as they like. Doctors can but protest—and submit to be called trade unionists for their pains! It is a queer world, my masters! The lunacy law is almost as mad as those with whom it deals. What more dangerous lunatic than the suspicious alcoholic? Yet the doctor who "certifies" him does so at his peril. Nothing happens, however, to the quack who sends him into solitary lodgings to commit suicide at his leisure.

### A Ridiculous Minister.

A man in a temper is always a ridiculous spectacle, and when it is a Secretary of State for the Home Office who, instead of giving careful attention to the inquiries of his subordinates and endeavouring to help them in their perplexities, flies into a tantrum and abuses other people, the spectacle is worse than ridiculous, for it tends to bring the Government into contempt and to give strength to the growing feeling of disgust with which the country regards the general ineptitude of the "official gang." A little time ago a case of criminal assault upon a child was brought before the magistrates at Bungay, and in the course of the evidence it turned out that so strong was the feeling among the medical men in the town as to the hardship entailed upon them by being dragged from their practices and kept hanging about assize courts for fees which are entirely insufficient, that it had been found impossible to induce any medical man in the town to examine the child. In view of the serious danger to the proper administration of justice involved in the continuance of such a state of affairs, the police authorities appealed to the Home Office for direction. Now one would have thought that this was a matter worthy of careful consideration, and of some judicious revision of the scale of fees which was fixed years and years ago, and even then was regarded as inadequate. But not at all. Mr. Ritchie must needs fly off at a tangent and abuse the doctors, speaking of their conduct as "very discreditable," even though in another sentence of the same letter he has to admit that they were perfectly within their rights in what they did; and then, as if he had not done enough to show his unfitness to deal with such questions, he must go on to display his ignorance of the very conditions of the problem by suggesting that if "in an exceptional case" in which there "might be danger of a serious miscarriage of justice"—miscarriages which are not "serious" we suppose do not matter—the police "should communicate with the Director of Public Prosecutions, who could"—not would—"if necessary employ an expert to make the examination." Fancy the police, when they require evidence as to conditions which would be likely to pass away in the course of a few hours, having to send their application through all the circumlocution of the Home Office. The whole thing is too absurd for words, and can only be explained on the ground that it is "official."

